# Physiological profile of adult male long-distance trail runners: variations according to competitive level (national or regional)

**DOI:** 10.31744/einstein_journal/2020AO5256

**Published:** 2020-03-30

**Authors:** Joana Oliveira-Rosado, João P. Duarte, Paulo Sousa-e-Silva, Daniela C. Costa, Diogo V. Martinho, Hugo Sarmento, João Valente-dos-Santos, Luís M. Rama, Óscar M. Tavares, Jorge Conde, Joaquim Castanheira, Rui Soles-Gonçalves, Ricardo R. Agostinete, Manuel J. Coelho-e-Silva

**Affiliations:** 1 Escola Superior de Tecnologia da Saúde de Coimbra Instituto Politécnico de Coimbra Coimbra Portugal Escola Superior de Tecnologia da Saúde de Coimbra, Instituto Politécnico de Coimbra, Coimbra, Portugal.; 2 Faculdade de Desporto Universidade do Porto Porto Portugal Faculdade de Desporto, Universidade do Porto, Porto, Portugal.; 3 Faculdade de Ciências do Desporto e Educação Física Universidade de Coimbra Coimbra Portugal Faculdade de Ciências do Desporto e Educação Física, Universidade de Coimbra, Coimbra, Portugal.; 4 Centro de Investigação do Desporto e da Atividade Física Universidade de Coimbra Coimbra Portugal Centro de Investigação do Desporto e da Atividade Física, Universidade de Coimbra, Coimbra, Portugal.; 5 Fundação para a Ciência e a Tecnologia Lisboa Portugal Fundação para a Ciência e a Tecnologia, Lisboa, Portugal.; 6 Faculdade de Educação Física e Desporto Universidade Lusófona de Humanidades e Tecnologia Lisboa Portugal Faculdade de Educação Física e Desporto, Universidade Lusófona de Humanidades e Tecnologia, Lisboa, Portugal.; 7 Laboratório de Investigação em Exercício Departamento de Educação Física Universidade Estadual Paulista “Júlio de Mesquita Filho” Presidente PrudenteSP Brazil Laboratório de Investigação em Exercício, Departamento de Educação Física, Universidade Estadual Paulista “Júlio de Mesquita Filho”, Presidente Prudente, SP, Brazil.; 8 Programa de Pós-graduação em Ciências da Motricidade Departamento de Educação Física Universidade Estadual Paulista “Júlio de Mesquita Filho” Presidente PrudenteSP Brazil Programa de Pós-graduação em Ciências da Motricidade, Departamento de Educação Física, Universidade Estadual Paulista “Júlio de Mesquita Filho”, Presidente Prudente, SP, Brazil.

**Keywords:** Physical exertion/physiology, Running, Exercise test, Oxygen consumption, Anaerobic threshold

## Abstract

**Objective:**

To describe and identify the importance of different indicators of the aerobic and anaerobic fitness of male ultra-trail runners according to their level of participation (regional or national).

**Methods:**

Forty-four male ultra-trail runners were assessed (36.5±7.2 years). They were classified as regional (n=25) and national (n=19). Wingate test was used to assess the anaerobic pathway. A progressive incremental running test was performed and ventilatory thresholds registered, in parallel to heart rate and lactate concentration at the end of the protocol. Comparison between groups was performed using independent samples *t*-test.

**Results:**

No significant differences were found between outputs derived from Wingate test. For aerobic fitness, while examining absolute values, differences were uniquely significant for the second ventilatory threshold (ultra-trail regional runners: 3.78±0.32L.min^-1^; ultra-trail national runners: 4.03±0.40L.min^-1^ p<0.05). Meantime, when aerobic fitness was expressed per unit of body mass, differences were significant for the second ventilatory threshold (ultra-trail regional runners: 50.75±6.23mL.kg^-1^.min^-1^; ultra-trail national runners: 57.88±4.64mL.kg^-1^.min^-1^ p<0.05) and also maximum volume of oxygen (ultra-trail regional runners: 57.33±7.66mL.kg^-1^.min^-1^; ultra-trail national runners: 63.39±4.26mL.kg^-1^.min^-1^ p<0.05).

**Conclusion:**

This study emphasized the importance of expressing physiological variables derived from running protocols per unit of body mass. Also, the second ventilatory threshold appears to be the best and the only aerobic fitness variable to distinguish between trail runners according to competitive level. Maximal oxygen uptake seems of relative interest to distinguish between long distance runners according to competitive level.

## INTRODUCTION

Trail running is generally performed on hiking trails with steep gradients, both uphill and downhill. Different from cross-country running, trail running is not listed among the International Association of Athletics Federation (IAAF) athletic disciplines. Trail running includes a wide range of distances, from short to ultra long (<42km and >100km, respectively)^(1)^ and has attracted a growing number of participants in recent years.^(2)^In spite of the increasing popularity of the sport, related research is still scarce.

Aerobic fitness seems an obvious determinant of performance in middle and long distance runners,^(3)^ even though maximal oxygen uptake is thought to be of minor importance in downhill sections.^(4)^ Variations in terrain characteristics in trial running competitions, particularly gradient variations, may place extraordinary demands on other metabolic pathways, such as the anaerobic. Percentage of maximum heart rate (HR) and oxygen uptake are subject to wide intra- and inter-individual variability in competitive sports activities. A recent study^(5)^described the physiological profile of runners taking part in a 65km mountain marathon, with 4,000m of cumulative elevation gain based on data collected from 23 amateur participants. Heart rate was monitored during the race and intensity zones were defined according to ventilatory threshold (VT) as zone I (<VT_1_), II (between VT_1_ and VT_2_) and III (>VT_2_). Mean race time in that study was 11.8 hours (±1.6 hour); mean race intensities were as follows: 85.7% zone I, 13.9% zone II and 0.4% zone III. Data related to exercise intensity variation are vital for competitive training and nutrition strategies.

Literature devoted to trail running addresses some topics, such as muscular performance,^(6)^ muscle damage,^(7)^ central fatigue and sleep deprivation^(8)^, as well as risk of musculoskeletal injuries.^(9)^ Yargic et al.,^(10)^ have recently examined the acute variation of molecules (interleukin − IL − 6, IL-15 and Hsp72) with significant effects on glucose and fat metabolism after a long distance trail run. Another recent study^(11)^ described biomechanical characteristics of short distance trail runners and their respective performances. Studies comparing ultra trail runners (UTRs) according to competitive level, if national (UTRs-N) or regional (UTRs-R), are lacking. Efficient conversion of metabolic energy into mechanical power is a major determinant of endurance performance.^(12)^ Mean HR intensity has been shown to progressively decline (running < cycling < swimming) in ultra-endurance triathlons.^(13)^ Competitions often have large withdrawal rates,^(14)^mainly due to inadequate pacing strategies (*i.e.,* choice of exercise intensity) or, in the case of trail runners, changes in energy costs associated with varying terrain (varying gradient in particular) interfering with running pace estimation.

An interesting intuitive question to be answered for endurance trail runners: “Why should fitness in prolonged endurance exercises, such as trail running, in which the oxygen uptake is not maximal, be determined by maximum volume of oxygen (VO_2peak_)”?

In the light of previous findings, this study set out to outline the profile of ultra trail runners competing at regional or national level, and to determine the significance of different indicators of aerobic and anaerobic fitness for differentiation of athletes according to competitive level. It was hypothesized that variables associated with metabolic pathways, other than VO_2_peak would be relevant to distinguish between long distance competing at different levels.

## OBJECTIVE

To describe and identify the importance of different indicators of the aerobic and anaerobic fitness of male ultra-trail runners according to their level of participation (regional or national).

## METHODS

### Study design and ethical requirements

Comparative cross-sectional study conducted at the Laboratory of Biokinetics located in the stadium of *Universidade de Coimbra*, in compliance with sports medicine ethical standards.^(15)^ This project was approved by the Ethics Committee of the *Universidade de Coimbra* (CE/FCDEF-UC/00102014). All participants were informed of study nature and objective and signed an Informed Consent Term. Participation was voluntary.

### Sample

Participants were recruited by convenience. Sample size was similar to that of previous studies with trail runners.^(1,6,10,11)^ The final sample comprised 44 adult male UTRs (mean chronological age: 36.5±7.2 years). Inclusion criteria were as follow: 2 or more years of participation and competition in the sport; having participated in regional or national competitions organized by the *Associação Trail Running Portugal*; and minimum of five competitions in the previous season. Runners with a history of loss time musculoskeletal injuries in the 2 months prior to the study were excluded. Participants were allocated to one of two groups according to competitive level: UTR-R and UTR-N. The UTR-R group comprised runners training with no professional guidance or advice to achieve competitive goals in the national ranking. The UTR-N group comprised runners training systematically under professional guidance and qualified for the national ranking. Ultra trail runners competing at the national level also competed in international events. Training experience data were collected by interviews.

### Anthropometry

Body mass and stature were measured to the nearest 0.1kg and 0.1cm using a scale (SECA balance, model 770, Hanover, MD, USA) and a stadiometer (Harpenden stadiometer, model 98.603, Holtain, Crosswell, UK), respectively.

### Aerobic fitness

Oxygen uptake data were collected during incremental treadmill (Quasar, HP Cosmos, Germany) running test. Flow and volume were calibrated using 3L syringe (Hans Rudolph, Kansas City, MO, USA) prior to each test; the gas analyzer (carbon dioxide and oxyen) (Quark, CPET, Cosmed, Italy) was calibrated using a kit (Cosmed, UN1956, 560L, 2200 psi, 70F). Air temperature and humidity were measured using a portable digital weather station equipped with thermo-hygrometer (Oregon Scientific, Model BAR913HGA, Tualatin, USA). All tests were performed at the same time of day (in the morning, between 10 a.m. and 12 p.m.). Final blood lactate level was measured using a portable analyzer (Lactate Pro2 Analyzer, Arcay, Inc.). Blood samples (25µL) were collected from the right thumb using a disposable lancet (UniStik 2 Extra). Samples were collected during the recovery period, immediately after maximum effort was reached. Athletes started the test running at 8km.h^-1^ at a constant gradient of 2% for 2 minutes. Running speed was increased by 1km.h^-1^ every minute to exhaustion; gradient was kept constant throughout.^(16)^ Heart rate was measured using a HR monitor (model T81 − CODED, Polar Electro, Finland). Peak oxygen uptake was defined by satisfaction of at least four out of five criteria, as follows: respiratory exchange ratio (RER) higher than 1.05; maximal HR over 95% of maximum value predicted for age; VO_2_ plateau in spite of increasing running speed; perception of exhaustion (Borg CR-10 Scale); blood lactate concentration >8mmol.L^-1^. The following variables were retained for subsequent analysis: VO_2_ values (VT_1_, VT_2_ and peak), maximal HR, RER and final lactatemia.

### Anaerobic fitness

The 30-second Wingate Anaerobic Test (WAnT) was conducted using a friction-loaded cycle ergometer (Monark AB, model 894E Peak Bike, Varberg, Sweden) connected to a microcomputer. The ergometer was calibrated according to manufacturer’s recommendations. Resistance was set at 0.075kg per unit of body mass. Participants were first submitted to a standardized 3-minute warm-up followed by a set of lower limb stretching exercises. Prior to countdown timer start, subjects pedaled at a constant rate of 60rpm with minimum resistance (basket supported). Standardized verbal encouragement was provided by observers. Wingate Anaerobic Test power outputs (WAnT peak, WAnT-P and WAnT mean, WAnT-M) were retained for analysis.

### Statistics analysis

Reliability of anthropometric measurements was determined according to technical error of measurement (TEM) and coefficients of variation (%CV). Anthropometric variables were measured twice in a subsample (n=13) for TEM determination; TEM was expressed in the same units and as percentage of the pooled mean (%CV), as follows: stature (TEM=0.37cm; %CV=0.21); body mass (TEM=0.56kg; %CV=0.81). This study involved two functional tests; therefore, quality control was not possible. Descriptive statistics were calculated for the overall sample (mean, standard error of the mean, 95% confidence interval, and standard deviation). Data normality was tested using the Kolmogorov-Smirnov test. Intergroup comparisons were based on the *t*- test for independent samples. The level of significance was set at 95%. Statistics analyses were performed using software (SPSS), version 25 for Windows; (SPSS Inc., IBM Company, Armonk, NY, USA). Figures were created using GraphPad Prism version 5.03 software (GraphPad Software, La Jolla, USA).

## RESULTS

**Figure 1:**
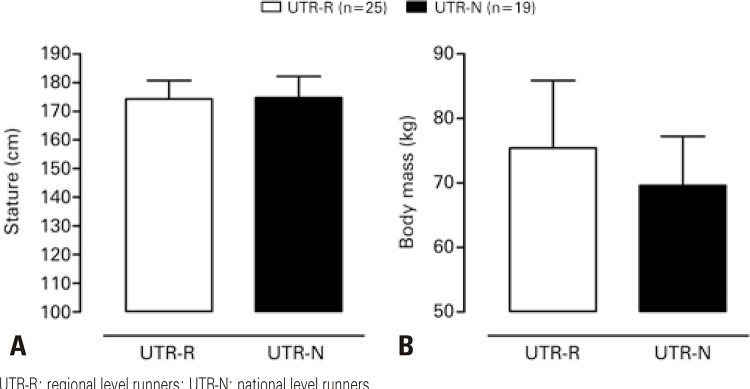
Mean stature and body mass by level of participation UTR-R: regional level runners; UTR-N: national level runners.

Descriptive characteristics of the sample (chronological age, training experience, stature, body mass, WAnT and aerobic test outputs) are summarized in table 1. All variables, except for training experience, body mass and RER, were marginally dependent variables according to study objectives. Briefly, sample characteristics were as follows: mean age of 36.5 years and 4 years of participation in the sport on average. Comparisons according to competitive level failed to reveal significant differences in training experience or stature between between UTRs-R and UTRs-N (Table 2). However, moderate mean body mass differences were noted, UTRs-R being 5.5kg heavier than UTRs-N (Figure 1).

**Table 1:** Descriptive statistics of the overall sample (n=44): chronological age, training experience, body size, Wingate test power outputs and data extracted from the incremental treadmill running test

Variable	Mean value	Standard error	95%CI	Standard deviation
Chronological age, year	36.5	1.1	34.3-38.7	7.2
Training experience, year	4.0	0.4	3.1-4.8	2.8
Stature, cm	174.4	1.0	172.3-176.5	6.9
Body mass, kg	73.0	1.5	70.1-76.0	9.6
WAnT-P, Watt	820	24	776-872	157
WAnT-M, Watt	587	13	562-615	86
Oxygen uptake: VT_1_, L.min^-1^	2.93	0.06	2.79-3.05	0.4
Oxygen uptake: VT_2_, L.min^-1^	3.87	0.06	3.75-4.00	0.4
Oxygen uptake: peak, L.min^-1^	4.32	0.06	4.21-4.43	0.4
Maximum heart rate, beats.min^-1^	175	1.5	172-178	9
RER, L.min^-1^ / L.min^-1^	1.16	0.01	1.14-1.16	0.1
Lactate, mmol.L^-1^	10.9	0.3	10.23-11.6	2.0

95%CI: 95% confidence interval;l WAnT-P: Wingate test peak output; WAnT-M: Wingate test mean output; VT_1_: first ventilatory threshold; VT_2_: second ventilatory threshold; RER: respiratory exchange ratio.

**Table 2:** Descriptive statistics (mean±standard deviation) according to competitive level and intergroup comparisons of chronological age, training experience, anthropometric data and outputs of functional tests assessing metabolic pathways

Dependent variables Y_i:_	X: independent variables		Comparison		
	
	Regional	National	Difference (95%CI)	Student *t* test	
		
	(n=25)	(n=19)		*t* value	p value
Chronological age, years	38.8±8.2	33.5±4.1	5.3 (1.5-9.1)	2.808	<0.01
Training experience, years	3.9±3.0	4.1±2.6	-0.2 (-1.9-1.6)	-0.199	0.84
Stature, cm	174.2±6.5	174.7±7.4	-0.5 (-4.8-3.7)	-0.243	0.81
Body mass, kg	75.4±10.5	69.9±7.6	5.5 (-0.2-11.2)	1.931	0.06
WAnT-P, Watt	816±164	824±١٥٠	-7.3 (-104.8-90.2)	0.151	0.88
WAnT-M, Watt	581±94	594±76	-13.6 (-66.8-39.6)	0.517	0.61
Oxygen uptake − VT_1_, L.min^-1^	2.95±0.32	2.96±0.48	-0.0 (-0.3-0.2)	-0.071	0.94
Oxygen uptake − VT_2_, L.min^-1^	3.78±0.32	4.03±0.40	-0.2 (-0.5- -0.0)	-2.238	0.03
Oxygen uptake − peak, L.min^-1^	4.26±0.35	4.40±0.36	-0.1 (-0.4-0.1)	-1.292	0.20
Maximum heart rate, beats.min^-1^	173±10	176±7	-3 (-9-2)	-1.360	0.25
RER	1.16±0.07	1.16±0.06	0.01 (-0.04-0.04)	-0.026	0.98
Lactate, mmol.L^-1^	11.42±1.83	10.27±2.04	1.1 (-0.2-2.5)	1.691	0.10
Oxygen uptake − VT_1_, mL.kg^-1^.min^-1^	39.56±5.15	42.45±5.73	-2.9 (-6.2-0.5)	-1.736	0.09
Oxygen uptake − VT_2_, mL.kg^-1^.min^-1^	50.75±6.23	57.88±4.64	-7.1 (-10.6- -3.7)	-4.156	<0.01
Oxygen uptake − peak, mL.kg^-1^.min^-1^	57.33±7.66	63.39±4.26	-6.1 (-9.8- -2.3)	-3.287	0.02

95%CI: 95% confidence interval; WAnT-P: Wingate test peak output; WAnT-M: Wingate test mean output; VT_1_: first ventilatory threshold; VT_2_: second ventilatory threshold; RER: respiratory exchange ratio.

**Figure 2:**
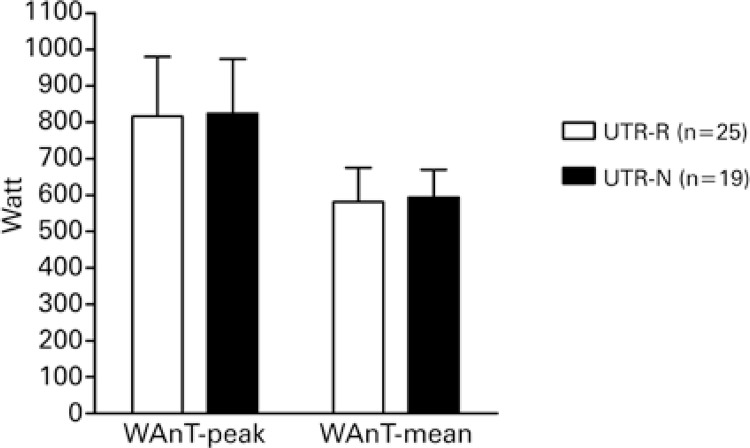
Mean values for anaerobic outputs obtained from the Wingate test by level of participation UTR-R: regional level runners; UTR-N: national level runners; WAnT-P: Wingate test peak output; WAnT-M: Wingate test mean output.

**Figure 3:**
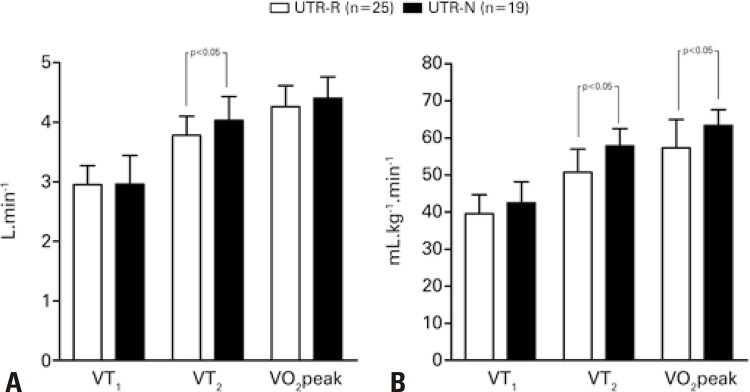
Mean values of variables measured during the treadmill running test expressed in absolute values and normalized for body mass, according to competitive level UTR-R: regional level runners; UTR-N: national level runners VT_1_: first ventilatory threshold; VT_2_: second ventilatory threshold; VO_2peak_: peak oxygen uptake.

Overall, UTRs-N tended to achieve higher absolute power outputs (WAnT; Figure 2), VTs and VO_2peak_ (Figure 3). Mean intergroup VT_2_ differences were only moderate (*t*=-2.238; p<0.05). However, following estimation of VT_2_ oxygen uptake values per unit of body mass, mean intergroup differences became large (*t*=-4.156; p<0.01). Differences were more or less pronounced depending on absolute or relative format of the parameters (absolute VT_2_: p<0.05 relative VT_2_: p<0.01) and VO_2peak_ values (absolute VO_2peak_: non-significant; relative VO_2peak_: p<0.05). Maximum HR, RER and blood lactate levels did not differ significantly between groups, in spite of mean differences of moderate magnitude in lactate levels, with lower values detected in the UTR-N group.

## DISCUSSION

This cross-sectional study outlined the profile of adult male UTR athletes according to competitive level (regional or national) based on results of different tests and variables associated with metabolic pathways. Major findings suggested runners in the UTR-N group were lighter in terms of absolute body mass and achieved better oxygen consumption values at intermediate variables VT_2_. No differences in peak oxygen uptake were found. However, oxygen uptake differences tended to be more pronounced when variables were expressed in relative (adjusted for body mass) compared to absolute values, suggesting a need for adequate weight management in long distance runners.

Differences detected at VT_2_ (absolute values) according to competitive level supported literature data. The second ventilator threshold (VT_2_), also named respiratory compensation point, is defined as the second breakpoint in ventilatory response due to acidosis (pH drop) caused by lactate production (insufficient bicarbonate buffering effect).^(17,18)^ Differences in absolute oxygen consumption between runners covering middle or long distances have been reported.^(19)^ Findings of this study suggest differences between UTRs competing at different levels may also reflect adaptability and can be in part explained by the fact that UTRs-N tend to be have access to more appropriate training programs, including bouts of acceleration and deceleration during the run, and are more often tested for morphological and physiological variables.

Lactate concentration and lactate threshold (markers of anaerobic metabolism contribution)^(20)^ are additional indicators of endurance performance evaluated in this study. Lower lactate levels during tests suggest higher ability of muscle tissues to prevent or delay the perception of fatigue, as rising blood lactate levels lead to premature exhaustion or muscle cramping, both of which are often associated with dropping out or poor performance in ultra-marathons.^(21)^ Low blood lactate levels in UTRs-N in this study reflected previous findings in middle and long distance runners.^(22)^ Less experienced long distance runners tend to adopt constant pace strategies^(5)^ to prevent lactate production, whereas runners with better competitive performances tend to run at varying pace and are able to recover better from short bouts of exercise out of their aerobic zone.

Wingate test power outputs (WAnT-P and WAnT-M) did not differ significantly between UTRs-N and UTRs-R in this study. The Wingate protocol is commonly used to estimate anaerobic fitness in several sport disciplines.^(22,23)^ Middle and long distance runners are known to achieve lower anaerobic power values compared to sprinters.^(12,24)^Despite training program differences, runners competing at the national or regional level in this study performed similarly in the Wingate test. Specific running protocols such as repeated sprints may be required to tease out potential differences between these two groups. In other words, being a cycle-ergometer-based test performed with subjects in the sitting position, the Wingate test may not be ideal to discriminate between UTRs.

This is the first study comparing metabolic pathways between UTRs according to competitive level (national or regional). However, some limitations need to be highlighted. First, voluntary recruitment of athletes yielded a small sample. Also, different (individually determined) initial speed may need to be adopted in protocols assessing aerobic fitness. Finally, length of exercise at each speed zone may not have been enough for oxygen uptake kinetics assessment. Future studies should also include blood sample collection after exercise completion at each speed zone.^(17)^

## CONCLUSION

Ultra distance trail running requires a well-trained aerobic component. Although maximal oxygen uptake is thought to be the best indicator of aerobic fitness, it did not come up as a vital component of aerobic fitness in this study. Ultra trail runners competing at different levels appeared to be more apt to vary their pace. Moderate to high values at the second ventilatory threshold seemed to be of benefit in extreme endurance activities. Trail runners competing at the national level achieved better outcomes at the second ventilatory threshold/respiratory compensation point, showing superior ability to respond to short bouts of higher intensity exercise. This versatility appeared to be specific to running, therefore this exercise pattern should be used to assess metabolic fitness in ultra distance trail runners instead of cycle-ergometer-based protocols. Finally, aerobic fitness in the sport in question appeared to be associated with optimal weight management, given differences between groups were more pronounced when physiological variables were expressed per unit of body mass. Future studies should account for intra-individual variation in physiological variables and competitive performance.
