# Supplementary material for: Physiological profile of adult male long-distance trail runners: variations according to competitive level (national or regional)
Source: Einstein (Sao Paulo). 2020 Mar 30;18:eAO5256. doi: 10.31744/einstein_journal/2020AO5256 (PMC7108817; doi:10.31744/einstein_journal/2020AO5256)
Supplement: Translation

# Translation: Perfil fisiológico de corredores de trilhas de longa distância adultos do sexo masculino: variações segundo o nível competitivo (nacional ou regional)

**Authors:** Joana Oliveira-Rosado, João P. Duarte, Paulo Sousa-e-Silva, Daniela C. Costa, Diogo V. Martinho, Hugo Sarmento, João Valente-dos-Santos, Luís M. Rama, Óscar M. Tavares, Jorge Conde, Joaquim Castanheira, Rui Soles-Gonçalves, Ricardo R. Agostinete, Manuel J. Coelho-e-Silva

**Affiliations:** 1 Portugal Escola Superior de Tecnologia da Saúde de Coimbra, Instituto Politécnico de Coimbra, Coimbra, Portugal.; 2 Portugal Faculdade de Desporto, Universidade do Porto, Porto, Portugal.; 3 Portugal Faculdade de Ciências do Desporto e Educação Física, Universidade de Coimbra, Coimbra, Portugal.; 4 Portugal Centro de Investigação do Desporto e da Atividade Física, Universidade de Coimbra, Coimbra, Portugal.; 5 Portugal Fundação para a Ciência e a Tecnologia, Lisboa, Portugal.; 6 Portugal Faculdade de Educação Física e Desporto, Universidade Lusófona de Humanidades e Tecnologia, Lisboa, Portugal.; 7 Brasil Laboratório de Investigação em Exercício, Departamento de Educação Física, Universidade Estadual Paulista “Júlio de Mesquita Filho”, Presidente Prudente, SP, Brasil.; 8 Brasil Programa de Pós-graduação em Ciências da Motricidade, Departamento de Educação Física, Universidade Estadual Paulista “Júlio de Mesquita Filho”, Presidente Prudente, SP, Brasil.

**Keywords:** Esforço físico/fisiologia, Corrida, Teste de esforço, Consumo de oxigênio, Limiar anaeróbio

## Abstract

**Objetivo:**

Descrever e comparar indicadores de aptidão metabólica em corredores de trilhas de longa distância (*ultra trail running*) adultos do sexo masculino, de acordo com o nível de competição (regional ou nacional).

**Métodos:**

Foram avaliados 44 corredores masculinos com média de idade de 36,5±7,2 anos classificados como de nível regional (n=25) ou nacional (n=19). Foi utilizado o teste de Wingate para avaliação da via anaeróbica, enquanto o teste incremental de corrida em esteira também foi realizado para determinar os limiares ventilatórios, o consumo máximo de oxigênio, a frequência cardíaca e a concentração de lactato ao final do protocolo. A comparação entre os grupos foi realizada por estatística teste *t* para amostras independentes.

**Resultados:**

As variáveis obtidas do teste Wingate não diferiram de forma significativa entre os grupos. No que diz respeito à aptidão aeróbica, foram encontradas diferenças significativas entre variáveis expressas em valores absolutos no segundo limiar ventilatório (corredores de nível regional: 3,78±0,32L.min^-1^; corredores de nível nacional: 4,03±0,40L.min^-1^; p<0,05). Quando considerados os valores expressos por unidade de massa corporal, o segundo limiar ventilatório (corredores de nível regional: 50,75±6,23mL.kg^-1^.min^-1^; corredores de nível nacional: 57,88±4,64mL.kg^-1^.min^-1^; p<0,05) e o volume máximo de oxigênio (corredores de nível regional: 57,33±7,66mL.kg^-1^.min^-1^; corredores de nível nacional: 63,39±4,26mL.kg^-1^.min^-1^; p<0,05) também diferiram de forma significativa.

**Conclusão:**

Este estudo destacou a importância de se expressarem variáveis fisiológicas derivadas de protocolos de corrida por unidade de massa corporal. Além disso, o segundo limiar ventilatório pareceu ser o melhor e único indicador de aptidão aeróbica para a diferenciação de corredores de trilha de longa distância, segundo o nível competitivo. O consumo máximo de oxigênio não é especialmente relevante para distinguir os corredores de trilha de longa distância, segundo o nível competitivo.

## INTRODUÇÃO

A corrida em todo terreno (ou em trilha, *trail run*) costuma ser praticada geralmente em locais montanhosos, com subidas e descidas e, ao contrário da corrida *cross-country*, não figura entre as modalidades reconhecidas pela *International Association of Athletics Federation* (IAAF). O esporte contempla uma ampla gama de distâncias, incluindo corridas curtas até ultralongas (<42km e >100km, respectivamente)^(1)^ e vem atraindo progressivamente maior número de participantes.^(2)^Pese a popularidade crescente do esporte, as pesquisas na área ainda são escassas.

A aptidão aeróbica parece ser um determinante óbvio do desempenho de corredores de meia e longa distância,^(3)^ embora o consumo máximo de oxigênio seja considerado menos importante nas descidas.^(4)^A variação de terreno, principalmente no que se refere ao grau de inclinação do percurso, pode impor grandes exigências sobre a via metabólica anaeróbica. A percentagem de utilização da frequência cardíaca (FC) máxima e o consumo de oxigênio sofrem substancial variação intra e interindividual em atividades esportivas de competição. Estudo recente^(5)^descreveu o perfil fisiológico de participantes de uma ultramaratona de montanha de 65km, com 4.000m de ganho acumulado (altitude), tendo por base dados relativos a uma amostra de 23 corredores amadores. A FC foi monitorada durante a corrida, e a intensidade foi definida de acordo com o limiar ventilatório (LV) como zonas I (<VT_1_), II (entre VT_1_ e VT_2_) e III (>VT_2_). O tempo médio de prova no estudo em questão foi de 11,8 horas (±1,6 hora), com as seguintes intensidades médias de corrida: 85,7% na zona I, 13,9% na zona II e 0,4% na zona III. Os dados referentes à variação de intensidade parecem fundamentais nas estratégias de competição e nos objetivos de treino, com implicações no que diz respeito às necessidades nutricionais.

A literatura dedicada ao esporte aborda alguns tópicos, como desempenho muscular,^(6)^lesão muscular,^(7)^ fadiga central e falta de sono,^(8)^ além de risco de lesões musculoesqueléticas.^(9)^ Recentemente, Yargic et al.,^(10)^ analisaram a variação aguda de moléculas (interleucina – IL − 6, IL-15 e Hsp72) dotadas de efeitos importantes sobre o metabolismo glicolítico e da gordura em corredores de trilhas de longa distância. Outro estudo^(11)^ descreveu as características biomecânicas de corredores de trilhas de curta distância e seus respectivos desempenhos. No entanto, faltam estudos comparando corredores de trilha de ultradistância (UTR - *ultra trail runners*) de acordo com os níveis competitivos nacional (UTR-N) ou regional (UTR-R). A eficiência na conversão de energia metabólica em potência mecânica é um dos principais determinantes do desempenho em esportes de longa duração (*endurance*).^(12)^ Nos triatlos de ultra *endurance*, as intensidades médias de FC decrescem progressivamente da corrida para o ciclismo para a natação.^(13)^ Não surpreendentemente, o índice de desistência em competições costuma ser alto^(14)^ principalmente devido a estratégicas inadequadas relativamente ao ritmo (isto é, seleção da melhor intensidade do exercício). No caso dos corredores de trilha, as variações de custo energético associadas às diferentes condições de terreno (principalmente à inclinação) dificultam a estimativa da velocidade de corrida.

Intuitivamente, parece ter interesse para corredores de trilha de longa distância saber o relevo do volume máximo de oxigênio (VO_2_max) para esforços prolongados de resistência, como a corrida em trilhas, nos quais o consumo de oxigênio não é máximo. À luz das informações disponíveis, este estudo pretende delinear o perfil de corredores de trilha que competem nas categorias regional ou nacional e, adicionalmente, determinar a importância de diferentes indicadores de aptidão aeróbica e anaeróbica na diferenciação dos atletas, de acordo com o nível competitivo. É colocada a hipótese de outros parâmetros marcadores da aptidão nas vias metabólicas, e não concretamente o VO_2_max, serem relevantes para a diferenciação de corredores de longa distância de acordo com o nível competitivo.

## OBJETIVO

Descrever e comparar indicadores de aptidão metabólica em corredores de trilhas de longa distância (*ultra trail running*) adultos do sexo masculino, de acordo com o nível de competição (regional ou nacional).

## MÉTODOS

### Desenho experimental e exigências éticas

Estudo comparativo transversal conduzido no Laboratório de Biocinética localizado no Estádio Universitário de Coimbra e em conformidade com os padrões éticos da medicina esportiva.^(15)^ Este projeto foi aprovado pelo Comitê de Ética da Universidade de Coimbra (CE/FCDEF-UC/00102014). A participação foi voluntária, e os participantes foram devidamente informados a respeito da natureza e dos objetivos do estudo. Todos os participantes assinaram um Termo de Consentimento Livre e Esclarecido.

### Amostra

Os participantes foram recrutados por conveniência. O tamanho amostral foi semelhante ao de estudos prévios com corredores de trilhas.^(1,6,10,11)^ A amostra final incluiu 44 UTRs adultos do sexo masculino (idade cronológica média: 36,5±7,2 anos). Os seguintes critérios de inclusão foram adotados: dois ou mais anos de prática e competição no esporte; ter participado de competições regionais ou nacionais organizadas pela Associação Trail Running Portugal; e com no mínimo cinco competições na temporada anterior. Corredores com histórico de lesão musculoesquelética e com perda de tempo de treino nos 2 meses que antecederam o estudo foram excluídos. A amostra foi dividida em dois grupos, segundo o nível competitivo: UTR-R e UTR-N. O grupo UTR-R incluiu corredores que não recebiam orientação formal de treinadores credenciados ou aconselhamento voltado para a conquista de objetivos competitivos no âmbito do *ranking* nacional. O grupo UTR-N incluiu corredores que treinavam sistematicamente sob orientação profissional e qualificados para o *ranking* nacional com participação em provas de âmbito internacional. Os dados referentes à experiência de treinamento foram coletados por meio de entrevistas.

### Antropometria

A massa corporal e a estatura foram mensuradas com margem de 0,1kg e 0,1cm empregando-se uma balança (SECA, modelo 770, Hanover, MD, USA) e um estadiômetro (Harpenden, modelo 98.603, Holtain, Crosswell, UK), respectivamente.

### Aptidão aeróbica

O consumo de oxigênio foi determinado por meio do teste incremental de esforço de corrida em esteira mecânica (Quasar, HP Cosmos, Germany). O fluxo e o volume foram calibrados empregando-se seringa de 3L antes de cada teste (Hans Rudolph, Kansas City, MO, USA). O analisador de gases (dióxido de carbono e oxigênio) (Quark, CPET, Cosmed, Italy) foi calibrado por meio de *kit* (Cosmed, UN1956, 560L, 2200 psi, 70F). A temperatura e a umidade do ar foram mensuradas empregando-se estação meteorológica portátil com termo-higrômetro (Oregon Scientific, Model BAR913HGA, Tualatin, USA). Todos os testes foram realizados na mesma hora do dia (pela manhã, entre 10 e 12 horas). O lactato final foi mensurado empregando-se analisador portátil (Lactate Pro2 Analyzer, Arcay, Inc.). Amostras de sangue (25µL) foram coletadas do polegar direito, empregando-se lanceta descartável (UniStik 2 Extra); as amostras foram coletadas durante o período de recuperação, imediatamente após o esforço máximo. Os atletas iniciaram o teste de esforço correndo por 2 minutos em velocidade de 8km.h^-1^, sob inclinação constante de 2%. Incrementos de velocidade de 1km.h^-1^ foram feitos a cada minuto até a exaustão, sem alteração da inclinação.^(16)^A FC foi registrada empregando-se monitor cardíaco (modelo T81 − CODED, Polar Electro, Finland). O consumo máximo de oxigênio foi definido pela observação de pelo menos quatro dos cinco critérios seguintes: taxa de troca respiratória (TTR) acima de 1,05; FC máxima acima de 95% do valor máximo estimado para a idade; *plateau* de VO_2_ apesar do aumento da velocidade de corrida; percepção de exaustão (escala CR-10 de Borg); e concentração sanguínea de lactato >8mmol.L^-1^. As seguintes variáveis foram retidas para análise subsequente: valores de VO_2_ (VT_1_, VT_2_ e valor máximo), FC máxima, TTR e lactatemia final.

### Aptidão anaeróbica

O teste anaeróbico de Wingate de 30 segundos (WAnT) foi realizado em um cicloergômetro com sistema de travagem de fricção (Monark AB, modelo 894E Peak Bike, Varberg, Suécia) conectado a um microcomputador. O ergômetro foi calibrado de acordo com as recomendações do fabricante. A resistência foi ajustada para 0,075kg por unidade de massa corporal. Os participantes foram inicialmente submetidos a um período de aquecimento de 3 minutos, seguido de uma série de exercícios de alongamento dos membros inferiores. Os participantes pedalaram a uma velocidade constante de 60rpm sob resistência mínima (*basket supported*) até o disparo do cronômetro. Durante o exercício, os participantes foram encorajados verbalmente pelos observadores. Os dados do teste anaeróbico de Wingate retidos neste estudo foram WAnT máximo (WAnT-Max) e WAnT médio (WAnT-Med).

### Análise estatística

A confiabilidade das medidas antropométricas foi determinada com base no erro técnico da medida (ETM) e nos coeficientes de variação (%CV). As variáveis antropométricas foram mensuradas duas vezes em uma subamostra (n=13) para determinação do ETM, expresso nas mesmas unidades de medida e também na forma de percentagem da média agrupada (%CV): estatura (ETM=0,37cm; %CV=0,21) e massa corporal (ETM=0,56kg; %CV=0,81). Dado o emprego de dois testes funcionais, não foi possível realizar controle de qualidade neste estudo. Parâmetros de estatística descritiva foram calculados para a amostra como um todo (média, erro padrão da média, intervalo de confiança de 95% e desvio padrão). A normalidade dos dados foi investigada empregando-se o teste de Kolmogorov-Smirnov. As comparações entre grupos foram baseadas no teste *t* para amostras independentes. O nível de significância considerado foi de 95%. As análises estatísticas foram realizadas por meio do programa (SPSS), versão 25 para Windows (SPSS Inc., IBM Company, Armonk, NY, USA). As figuras foram criadas a partir do programa GraphPad Prism versão 5.03 (GraphPad Software, La Jolla, USA).

## RESULTADOS

As características descritivas da amostra (idade cronológica, experiência de treinamento, estatura, massa corporal, resultados do WAnT e do teste aeróbico) foram resumidas na tabela 1. Todas as variáveis, exceto a experiência de treinamento, massa corporal e TTR, mostraram-se marginalmente dependentes com relação aos objetivos do estudo. Resumidamente, a amostra apresentou as seguintes características: média de idade de 36,5 anos e 4 anos de participação no esporte. As comparações de acordo com o nível competitivo não indicaram diferenças significativas entre UTRs-R e UTRs-N no que se refere à experiência de treinamento e à estatura (Tabela 2). Por outro lado, foram observadas diferenças médias moderadas de massa corporal, sendo os UTRs-R 5,5kg mais pesados do que os UTRs-N (Figura 1).

**Tabela 1:** Estatística descritiva dos dados da amostra (n=44): idade cronológica, experiência de treinamento, tamanho corporal, potência atingida no teste Wingate e resultados do teste incremental de corrida em esteira

Variável	Valor médio	Erro padrão	IC95%	Desvio padrão
Idade cronológica, anos	36,5	1,1	34,3-38,7	7,2
Experiência de treinamento, anos	4,0	0,4	3,1-4,8	2,8
Estatura, cm	174,4	1,0	172,3-176,5	6,9
Massa corporal, kg	73,0	1,5	70,1-76,0	9,6
WAnT-Max, Watt	820	24	776-872	157
WAnT-Med, Watt	587	13	562-615	86
Consumo de oxigênio: LV_1_, L.min^-1^	2,93	0,06	2,79-3,05	0,4
Consumo de oxigênio: LV_2_, L.min^-1^	3,87	0,06	3,75-4,00	0,4
Consumo de oxigênio: máximo, L.min^-1^	4,32	0,06	4,21-4,43	0,4
Frequência cardíaca máxima, batidas.min^-1^	175	1,5	172-178	9
TTR, L.min^-1^ / L.min^-1^	1,16	0,01	1,14-1,16	0,1
Lactato, mmol.L^-1^	10,9	0,3	10,23-11,6	2,0

IC95%: intervalo de confiança de 95%; WAnT-Max: potência máxima no teste Wingate; WAnT-Med: potência média no teste Wingate; LV_1_: primeiro limiar ventilatório; LV_2_: segundo limiar ventilatório; TTR: taxa de troca respiratória.

**Tabela 2:** Estatística descritiva (média±desvio padrão) por nível competitivo e comparações entre grupos dos dados de idade cronológica, experiência de treinamento, variáveis antropométricas e resultados dos testes funcionais empregados na avaliação das vias metabólicas

Variáveis dependentes Y_i:_	X: variáveis independentes		Comparação		
	
	Regional	Nacional	Diferença (IC95%)	Teste *t* de Student	
		
	(n=25)	(n=19)		Valor de *t*	Valor de p
Idade cronológica, anos	38,8±8,2	33,5±4,1	5,3 (1,5-9,1)	2,808	<0,01
Experiência de treinamento, anos	3,9±3,0	4,1±2,6	-0,2 (-1,9-1,6)	-0,199	0,84
Estatura, cm	174,2±6,5	174,7±7,4	-0,5 (-4,8-3,7)	-0,243	0,81
Massa corporal, kg	75,4±10,5	69,9±7,6	5,5 (-0,2-11,2)	1,931	0,06
WAnT-Max, Watt	816±164	824±١٥٠	-7,3 (-104,8-90,2)	0,151	0,88
WAnT-Med, Watt	581±94	594±76	-13,6 (-66,8-39,6)	0,517	0,61
Consumo de oxigênio − LV_1_, L.min^-1^	2,95±0,32	2,96±0,48	-0,0 (-0,3-0,2)	-0,071	0,94
Consumo de oxigênio − LV_2_, L.min^-1^	3,78±0,32	4,03±0,40	-0,2 (-0,5- -0,0)	-2,238	0,03
Consumo de oxigênio − máximo, L.min^-1^	4,26±0,35	4,40±0,36	-0,1 (-0,4-0,1)	-1,292	0,20
Frequência cardíaca máxima, batidas.min^-1^	173±10	176±7	-3 (-9-2)	-1,360	0,25
TTR	1,16±0,07	1,16±0,06	0,01 (-0,04-0,04)	-0,026	0,98
Lactato, mmol.L^-1^	11,42±1,83	10,27±2,04	1,1 (-0,2-2,5)	1,691	0,10
Consumo de oxigênio − LV_1_, mL.kg^-1^.min^-1^	39,56±5,15	42,45±5,73	-2,9 (-6,2-0,5)	-1,736	0,09
Consumo de oxigênio − LV_2_, mL.kg^-1^.min^-1^	50,75±6,23	57,88±4,64	-7,1 (-10,6- -3,7)	-4,156	<0,01
Consumo de oxigênio − máximo, mL.kg^-1^.min^-1^	57,33±7,66	63,39±4,26	-6,1 (-9,8- -2,3)	-3,287	0,02

IC95%: intervalo de confiança de 95%; WAnT-Max: potência máxima no teste Wingate; WAnT- Med: potência média no teste Wingate; LV_1_: primeiro limiar ventilatório; LV_2_: segundo limiar ventilatório; TTR: taxa de troca respiratória.

**Figura 1:** Estatura e massa corporal médios de acordo com o nível competitivo UTR-R: corredores de nível regional; UTR-N: corredores de nível nacional.

De forma geral, os UTRs-N tenderam a atingir melhores valores absolutos de potência (WAnT; Figura 2), LVs e VO_2max_ (Figura 3). As diferenças médias de LV_2_ entre os grupos foram apenas moderadas (*t*=-2,238; p<0,05). Entretanto, após o ajuste dos valores de consumo de oxigênio no LV_2_ para unidade de massa corporal, as diferenças entre os grupos tornaram-se grandes (*t*=-4,156; p<0,01). Na verdade, as diferenças foram mais pronunciadas dependendo do formato com que foram expressos os parâmetros ventilatórios (VT_2_ absoluto: p<0,05 VT_2_ relativo: p<0,01) e VO_2max_ (VO_2max_ absoluto: não significativo; VO_2max_ relativo: p<0,05). A FC máxima, a TTR e o lactato sanguíneo não diferiram de forma significativa entre os dois grupos, apesar das diferenças médias de magnitude moderada na concentração de lactato, com valores mais baixos detectados no grupo UTR-N.

**Figura 2:** Valores médios de potência obtidos no teste Wingate de acordo com o nível competitivo UTR-R: corredores de nível regional; UTR-N: corredores de nível nacional; WAnT-Max: potência máxima no teste Wingate; WAnT-Med: potência média no teste Wingate.

**Figura 3:** Valores médios das variáveis mensuradas durante o teste de esteira expressos em valores absolutos e normalizados para massa corporal, de acordo com o nível competitivo UTR-R: corredores de nível regional; UTR-N: corredores de nível nacional; LV_1_: primeiro limiar ventilatório; LV_2_: segundo limiar ventilatório; VO_2max_: volume máximo de oxigênio.

## DISCUSSÃO

Este estudo transversal delineou o perfil de atletas adultos do sexo masculino praticantes de UTR de acordo com o nível competitivo (regional ou nacional), com base nos resultados de diferentes testes e em variáveis associadas às vias metabólicas. Os principais resultados sugerem que corredores do grupo UTR-N apresentaram menor massa corporal em valores absolutos e obtiveram melhores valores de consumo de oxigênio nas variáveis intermediárias do LV_2_. Os valores de consumo máximo de oxigênio não diferiram. Entretanto, as variáveis de consumo de oxigênio tenderam a diferir de forma mais pronunciada quando expressas em valores relativos (ajustados para massa corporal) do que quando expressas em valores absolutos, sugerindo a necessidade de controle adequado do peso corporal em corredores de longa distância.

As diferenças detectadas no LV_2_ (valores absolutos) de acordo com o nível competitivo corroboram os dados disponíveis na literatura. O LV_2_, também chamado de ponto de compensação respiratória, é definido como o segundo ponto de quebra na resposta ventilatória em decorrência da acidose (queda do pH) causada pela produção de lactato (efeito insuficiente de tamponamento pelo bicarbonato).^(17,18)^Diferenças de consumo absoluto de oxigênio entre corredores de distâncias médias ou longas já foram relatadas.^(19)^ Os dados deste estudo sugerem ainda que as diferenças detectadas entre UTRs de níveis competitivos contrastantes também podem refletir adaptabilidade, podendo ser, em parte, explicadas pelo fato de UTRs-N terem acesso a programas mais adequados de treinamento, incluindo acelerações e desacelerações rápidas ao longo da corrida, além de serem submetidos a avaliações de parâmetros morfológicos e fisiológicos com maior frequência.

Outros indicadores de desempenho em atividades de resistência utilizados neste estudo foram o limiar e a concentração de lactato (marcadores da prova na esteira para a via anaeróbica).^(20)^ Concentrações mais baixas de lactato durante os testes sugerem maior capacidade do músculo de prevenir ou protelar a percepção de fadiga, uma vez que o acúmulo de lactato sanguíneo leva à exaustão precoce ou ao desenvolvimento de câimbras, fatores esses associados à desistência ou ao mau desempenho em ultramaratonas.^(21)^ A baixa concentração sanguínea de lactato detectada nos UTRs-N neste estudo corrobora estudos prévios em corredores de distâncias médias e longas.^(22)^ Corredores de longa distância menos experientes tendem a manter um ritmo constante de corrida^(5)^ para evitar a produção de lactato, enquanto corredores com desempenho competitivo melhor tendem a variar o ritmo e apresentam melhor recuperação após episódios curtos de exercício fora da zona aeróbica.

No presente estudo, os níveis de potência atingidos no teste Wingate (WAnT-Max e WAnT-Med) não diferiram de forma significativa entre UTRs-N e UTRs-R. O protocolo Wingate é frequentemente utilizado para se estimar a aptidão anaeróbica em diversos esportes.^(22,23)^ Sabe-se que corredores de médias e longas distâncias atingem valores menores de potência anaeróbica do que corredores de velocidade.^(12,24)^Apesar das diferenças de treinamento entre corredores classificados para competir no nível nacional ou regional, o desempenho de ambos os grupos no teste Wingate foi semelhante neste estudo. A investigação de diferenças entre os dois grupos talvez reclame protocolos específicos de corrida, como tiros repetidos em alta velocidade. Em outras palavras, sendo um teste realizado em cicloergômetro, com o participante na posição sentada, o teste Wingate pode não ser ideal para discriminar UTRs.

Este estudo, ao comparar vias metabólicas entre UTRs de acordo com o nível competitivo (nacional ou regional), contribui para o conhecimento disponível em ciências do desporto. Entretanto, algumas limitações devem ser ressaltadas. Em primeiro lugar, o recrutamento voluntário de atletas gerou uma amostra pequena. Além disso, protocolos de avaliação de aptidão aeróbica que adotem velocidades iniciais diferentes (determinadas individualmente) podem ser necessários. Finalmente, é possível que a duração do exercício em cada patamar de velocidade não tenha sido suficiente para a avaliação da cinética do consumo de oxigênio. Estudos futuros também devem incluir coletas de sangue ao final do exercício em cada patamar de velocidade.^(17)^

## CONCLUSÃO

Os treinos e as competições de corrida de ultradistância em trilhas exigem um componente aeróbico bem desenvolvido. Embora o consumo máximo de oxigênio seja considerado o melhor indicador de aptidão aeróbica, o mesmo não se confirmou como componente fundamental da aptidão da via aeróbica. Corredores de trilhas de ultradistância que competem em diferentes níveis parecem ter maior capacidade de ajustar o ritmo de corrida. Valores moderados a altos no segundo limiar ventilatório se mostraram vantajosos para a prática de atividades de resistência extrema. Corredores de trilha que competem no nível nacional obtiveram melhores resultados no segundo limiar ventilatório/ponto de compensação respiratória, o que sugere maior capacidade de resposta a episódios curtos de exercício de alta intensidade. Essa versatilidade mostrou-se específica para a corrida, sendo este o padrão de esforço de eleição para avaliação da aptidão metabólica em corredores de trilha de ultradistância, e não os protocolos baseados cicloergômetros. Finalmente, a aptidão aeróbica no esporte agora estudado mostrou-se associada à manutenção do peso corporal ideal, visto que as diferenças entre os grupos surgiram mais pronunciadas quando os parâmetros fisiológicos foram expressos por unidade de massa corporal. Novos estudos devem levar em conta a variação intraindividual de variáveis fisiológicas e desempenho competitivo.
